# Combined Chair-Based Exercises Improve Functional Fitness, Mental Well-Being, Salivary Steroid Balance, and Anti-microbial Activity in Pre-frail Older Women

**DOI:** 10.3389/fpsyg.2021.564490

**Published:** 2021-03-25

**Authors:** Guilherme Eustáquio Furtado, Rubens Vinícius Letieri, Adriana Silva-Caldo, Joice C. S. Trombeta, Clara Monteiro, Rafael Nogueira Rodrigues, Ana Vieira-Pedrosa, Marcelo Paes Barros, Cláudia Regina Cavaglieri, Eef Hogervorst, Ana Maria Teixeira, José Pedro Ferreira

**Affiliations:** ^1^Research Unit in Sport and Physical Activity-CIDAF (UID/PTD/04213/2020), Faculty of Sport Sciences and Physical Education (FCDEF-UC), University of Coimbra, Coimbra, Portugal; ^2^Health Sciences Research Unit: Nursing (UICISA:E), Nursing School of Coimbra (ESEnfC), Coimbra, Portugal; ^3^Rehabilitation Sciences Program, Federal University of Alfenas (UNIFAL), Alfenas, Brazil; ^4^Multidisciplinary Research Nucleus in Physical Education (NIMEF), Physical Education Department, Federal University of Tocantins (UFT), Tocantinópolis, Brazil; ^5^Exercise Physiology Laboratory (FISEX), Faculty of Physical Education, University of Campinas (UNICAMP), Campinas, Brazil; ^6^Cardiopulmonary Physiotherapy Laboratory, Federal University of São Carlos (UFscar), São Carlos, Brazil; ^7^Institute of Physical Activity Sciences and Sports (ICAFE), Interdisciplinary Program in Health Sciences, Cruzeiro do Sul University, São Paulo, Brazil; ^8^Graduate Program in Gerontology, Faculty of Medical Sciences, University of Campinas (UNICAMP), Campinas, Brazil; ^9^Applied Cognitive Research NCSEM, Loughborough University, Loughborough, United Kingdom

**Keywords:** frailty, subjective well-being, aging, health, cortisol, testosterone, immune system

## Abstract

**Introduction:**

Regular exercise has long been shown to positively impact the immune system responsiveness and improve mental well-being (MWB). However, the putative links between biomarkers of mental health and immune efficiency in exercising subjects have been scarcely investigated. The aim of this study was to verify the effect of a 14-week combined chair-based exercise program (CEP) on salivary steroid hormones and anti-microbial proteins, functional fitness, and MWB indexes in pre-frail older women.

**Methods:**

The participant women (82.8 4.6 years old; *n* = 32) were randomly divided into the exercising group (CEP, *n* = 17) and the non-exercising control group (CG, *n* = 15). The pre/post assessment included: (1) salivary anti-microbial proteins lysozyme; (Lys) and immunoglobulin-A (IgA); (2) salivary steroid hormones of testosterone (TT) and cortisol (COR); (3) functional fitness (gait speed, hand grip strength, and static balance); (4) MWB questionnaires (happiness, depression state, satisfaction with life, and stress).

**Results:**

Significant differences with large Cohen’s (*d*) effect sizes were found on increased salivary TT (*p* < 0.05; *d* = 0.60) after exercise intervention. The results revealed a decrease in IgA levels after CEP (*p* < 0.01, *d* = 0.30). The increase in subjective happiness levels (*p* < 0.05, *d* = 0.30) and decrease of stress perception (*p* < 0.01, *d* = 2.60) and depressive state (*p* < 0.05, *d* = 0.30) were found after intervention in the CEP group. Robust statistical differences in gait speed (*p* < 0.05; *d* = 0.60) and balance tests (*p* < 0.05; *d* = 0.80) were also found in the CEP group. In control, COR increased moderately (*p* < 0.05; *d* = 0.65) while no changes were found for the other indicators. Correlation analyses showed inter-dependence between pre–post variations of MWB, biochemical indexes, and fitness function (e.g., COR inverse correlation with hand grip strength and balance tests).

**Conclusion:**

The CEP program was able to improve functional-fitness performance, decrease feelings of stress, and increase happiness. The CEP also induced clinically relevant hormonal and immune responses, which suggests that chair exercises that combine muscular strength, balance, and gait speed training are promising interventions to improve physical and mental health of older pre-frail adults.

## Introduction

Aging is a natural progressive process of morphologic and physiologic alterations that innately predisposes older populations to a gradual poor health regression (Clegg et al., 2013). Despite the natural decline of some cognitive and physiological functions with aging (Gruver et al., 2007), concomitant harmful factors, such as malnutrition, lack of physical activity, social isolation, depression, etc., could exacerbate these dysfunctions, aggravating the mental and physical adverse health conditions of older adults (Artaza-Artabe et al., 2016). This dysfunctional cognitive-physical state is called cognitive frailty, which is also recognized by the general vulnerability in offering a prompt homoeostatic response after a stressor episode, and are thought to be the result of cumulative weakening of many cognitive and psychophysiological functions throughout a lifecycle (Ruan et al., 2015).

Regarding the mechanisms of physiological responses to stress, it is well known that the hypothalamic–pituitary–adrenal (HPA) axis is highly responsive to emotional and environmental stress, displaying cortisol (COR) and testosterone (TT) as main protagonists of the psychosomatic effects of stress, especially via the autonomic nervous system (Hek et al., 2013). The exposure to chronic stressors, and consequently the hyperactivation of physiological stress systems, will increase heart rate and basal oxygen uptake, elevate COR, TT, and other steroid hormone levels (to induce endocrine imbalances) (Rhebergen et al., 2015), interfere in energy metabolism (with putative induction of metabolic disorders, such as obesity and related diabetes), hinder immune responses, and inhibit organism defensive systems (Baylis et al., 2013). Altogether, these factors will contribute to accelerate biological aging, often associated with severe comorbidities and frailty (Révész et al., 2014).

Among several non-pharmacological strategies to treat frailty, combined muscle strength and aerobic exercises–although still dependent on an adequate nutrition (Artaza-Artabe et al., 2016)–have been shown as the easiest and most cost-effective intervention to delay or reverse frailty to implement in primary care (Travers et al., 2019). Timely diagnosis and interventions to address frailty is essential for older individuals to build resilience and live independently, but also help health systems use resources more efficiently in the context of growing life expectancy worldwide (Park and Lee, 2010; Clegg et al., 2012; Jadczak et al., 2018).

The positive effects of regular exercise are extended throughout many biological levels in the practitioners, including metabolic adaptations (Barbosa et al., 2009), such as e.g., induction of muscle lactate dehydrogenase and tricarboxylic acid cycle enzymes, hepatic gluconeogenesis, more efficient protein turnover, etc., physiological gains (especially skeletal-contractile, cardiovascular, and respiratory capacities), adjustment of hormonal balance (glycemic glucagon/insulin ratio, TT and COR levels, etc.), and cognitive-psychological benefits, such as good mood, higher well-being perception, anxiolytic and anti-depressive effects, and others (Travers et al., 2019). Regarding older adults, all these benefits are strongly recommended to promote a safe, independent and physically–mentally healthy life (Walsh et al., 2011; Hogervorst and Clifford, 2012; Hatta et al., 2013).

Taking the physical limitations of frail (and pre-frail) older individuals, exercise adaptations and special training protocols have been suggested (Doody et al., 2019; Grimmer et al., 2019). Among many adapted protocols, chair-seated exercises have gained much interest nowadays since it imposes an autonomous resistance effort concomitantly avoiding risks of injuries and high impact on the articulations of practitioners (Rathleff et al., 2017; Furtado et al., 2020). Thus, the aim of this study is to verify the effect of a chair-based combined exercise program (CEP) on salivary COR, TT, and biomarkers of anti-microbial activity [immunoglobulin-A (IgA) and lysozyme (Lys), respectively], their link to functional status, and positive and negative mental well-being (MWB) in pre-frail older women.

## Materials and Methods

### Initial Procedures

Older adult women (≥65 years) were recruited to participate in this study. The participants were residents of social and health care support centers from Coimbra, Portugal, and were part of a more comprehensive study protocol recently carried out by our group (Teixeira et al., 2016). Participants and their guardians were required to give a full informed written consent before beginning the research. This study was approved by the Faculty of Sport Sciences and Physical Education Ethical Committee–University of Coimbra reference code CE/FCDEF-UC/000202013; it respects the Portuguese Resolution (Art.° 4st; Law n. 12/2005, 1st series) on ethics in research with humans (Braga, 2013), follows the guidelines for ethics in scientific experiments in exercise science research (Shephard, 2002), and complies with the guidelines for research with human beings of the Helsinki Declaration (Petrini, 2014). This clinical trial is officially registered at ClinicalTrials.gov with the registration ID: NCT04435899.

### Study Design

**FIGURE 1:**
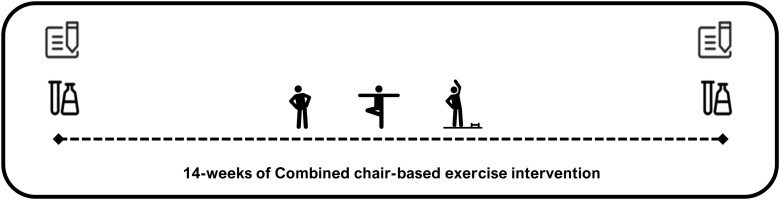
Graphical scheme of intervention study design.

This is an interventional pre–post randomized (controlled) trial study that investigated the effects of a 14-week combined chair-based exercise program (CEP) on salivary immune biomarkers, functional fitness, and happiness–well-being perception in institutionalized pre-frail older women. Our hypothesis is that combined chair-based exercises will result in both physical (immunological and functional fitness) and mental improvements in pre-frail women, to bring them more autonomy and increase their quality of life. The physical and psychological tests were applied to all groups before and after (pre/post) the exercise intervention (Figure 1).

### Sample Selection Criteria

Inclusion criteria were as follows: (i) women should be aged over 65 years; (ii) if dependent of drug therapy, it should be controlled and updated; (iii) if the participant presented a clinical condition or comorbidity, it must be stable and medicated (as shown in item ii); and (iv) they should be physically able to participate in exercise classes, based on local medical diagnosis. The Exclusion Criteria were (i) participating in other structured exercise programs; (ii) presenting severe cardiomyopathy, asthmatic bronchitis or uncontrolled hypertension, musculoskeletal disorders that limit physical tests (i.e., osteoarthritis, recent fractures), psychiatric disorder or dementia (e.g., diagnosed severe cognitive impairment or Alzheimer), hearing and vision impairment, morbid obesity or the use of medications that significantly affect attention; and (iii) adherence to the exercise program ≤60%. In addition, biosocial, social interactive behavior, and overall health status, evidenced by the local clinical staff reports, were also included as a post-inclusion criteria to finalize the selected group of participants.

### Participants

The initial sample was composed of 60 institutionalized women, who were mainly sedentary from two social and health care institutions. We also recruited 18 additional participants to avoid an estimated loss of 30% of the participants during the study, based on previous studies from our group (Rieping et al., 2019). Accordingly, we counted 47 participants at the end of the intervention (age = 82.8 ± 4.6 years) who were randomly assigned to one of two groups using software (Randomizer App, V-team ESRB): the combined CEP (*n* = 17) and the non-exercising control group (CG) (*n* = 15) who received care as usual. The sample size was calculated using G^∗^Power (version 3.1.9.2). Alpha was adjusted at 0.05 and power at 0.85 to allow for repeated measures ANOVA. A 14-week period was applied for the CEP intervention. The CG group did not participate in any type of supervised exercise intervention, but was encouraged to engage in complementary activities provided by the institutions, like outside tours, art education, and cultural activities, as well as maintaining their regular daily activities during the 14-week period (see Figure 1).

### Comorbidities

The Charlson Comorbidity index (CCI) was used to identify possible comorbidities. This method predicts the levels of comorbidities and mortality by classifying (or weighting) comorbid conditions. This instrument has been widely used by health researchers to evaluate burden of disease and has a weighted index based on 17 comorbid conditions, which has been shown to predict 1- and 10-year mortality (Quan et al., 2011).

### Combined Chair-Based Exercises

To create a progressive CEP to improve the walking capability, balance, and muscle strength and resistance, specific numbers of exercises (7–10) were performed with a determined number of repetitions (6–10), sets (2–3), cadence of execution (1:2), and rest between sets (45–60 s), following a circuit training protocol (Giné-Garriga et al., 2014). In addition, the CEB method was integrated in this program. The CEB consists of systematized and gradual exercises performed with a chair for support that guarantees the individual’s stability during the session, respecting individual limitations without discouraging individuals to reach beyond their limits (Kevin et al., 2011). However, the goal is to decrease the time of using the chair support, aiming to increase the standing position during the sessions. Intensity was indirectly calculated using Karvonen’s formula to predict target heart rate (HR). The maximum HR (HR_max_) was calculated using a specific formula for older population (Tanaka et al., 2001): [Target Heart Rate = [(HR_max_ – resting HR) × %Intensity] + resting HR]. The HR_max_ was monitored using heart rate monitors (Polar, RCX5) randomly distributed among participants. A low to moderate intensity effort, around 50–75% of maximum heart rate zone (HRz_max_), was attained as recommended by the ACSM (Nelson et al., 2007). In addition, intensity was measured by the modified BORG scale of perceived exertion (PSE), that consists of an arbitrary scale ranging from 0 to 10 points (pts), with identical intervals and with reference to the quality of effort: (0) nothing at all; (1) very weak; (2) weak; (3) moderate; (4) somewhat strong; (5–6) strong; (7–9) very strong; (10) very, very strong (almost maximal). Each session was divided into five parts: 7 min of warm-up and body mobilization (PSE = 1–3, HRz_max_ = 50–55%); 15 min of low/upper body elastic-band exercises, 15 min of static and dynamic balance exercises, 15 min of sequential exercises improving gait speed (PSE 3–4, HRz_max_ = 56–70%) and finally, 7 min of stretching exercises as a “cool down” strategy (PSE 1–2, HRz_max_ = 45–50%). The frequency of classes was 2–3 times/week, for 14 weeks, to totalize 32 sessions and attendance was documented daily.

### Assessments

The experimental approach collected information on the global health and biosocial status of the participants, applied the validated Portuguese version of psychometric rate scales for screening MWB, assessed the functional fitness of participants, measured the anthropometric indexes, and determined the salivary steroid hormones and anti-microbial protein levels. All data were collected and processed by expert technicians and trained researchers.

### Frailty Status

Physical frailty (PF) was assessed using the hand grip strength test (HGT), following the criteria of the Fried protocol (Fried et al., 2001). Recent findings demonstrate that HGT is a useful single marker for frailty status screen (Syddall et al., 2003). The HGT test uses a hand-held dynamometer (HD), and strength kilograms is a unit of measure (Lafayette Dynamometer, model 78010, United States). The participants hold the HD in the dominant hand to be tested, with their elbow by the side of the body. When ready, the participant squeezes the HD with the highest isometric effort, which is sustained for 5 s. The best score of three trials was used for scoring purposes. The selected score was adjusted by gender and body mass index. In the case of this study, the cut-off value of BMI 23–28 (HGT scores 15–18 kg) was used for screen pre-frail individuals.

### Salivary Biomarkers

Saliva collection was carried out in the morning (between 9:00 and 11:30 a.m.), at least 30 min after the first diurnal food intake. The participants remained seated, with their head slightly tilted down, eyes open, and oriented to perform a minimum of orofacial movements. Saliva samples were collected in polypropylene tubes, then sealed, and immediately refrigerated at −20°C. The levels of COR and TT in saliva were measured by competitive ELISA (kit #1-3002 and #1-2402, respectively; Salimetrics, United Kingdom). The concentrations of Lys and IgA in saliva were also determined by ELISA (respectively, kit ab108880, Abcam, United Kingdom; and #1-1602, Salimetrics, United Kingdom). The determination of salivary markers followed the manufacturer instructions and were described in a previous study (Allgrove et al., 2008). The sensitivity and range of detection limits for COR (<0.007 and 0.012–3.000 μg/dl), TT (1.0 and 6.1–300 pg/ml), Lys (0.1 and 0–300 μg/dl), and IgA (2.5 and 2.5–100 μg/dl) were reported by the manufacturer (Miller et al., 2013).

### Global Health and Biosocial Status

Clinical and sociodemographic information was also collected: age, sex, marital status, and education. In addition, the comorbidity index was applied to screen the clinical history related to chronic diseases and cognition profile of the participants with the help of the institutional medical staff.

### Anthropometric Measurements

The anthropometric measurements were performed following standardized procedures (Baumgartner et al., 1989). Body mass was measured (kg) using a portable scale (Seca, model 770, Germany) with a precision of 0.1 kg. Waist circumference was measured using a retractable glass fiber tape measure (Hoechstmass-Rollfix, Germany) with a precision of 0.1 cm. Stature was determined using a portable stadiometer (Seca Bodymeter, model 208, Germany) with a precision of 0.1 cm.

### Nutritional Status

The nutritional status of participants was assessed using the Mini-Nutritional Assessment questionnaire (MNA). This is an 18-item questionnaire that includes four domains, namely, anthropometric, general health, dietary, and self-assessment of health and nutritional status. The maximum score of MNA is 30 pts, and classifies subjects as well-nourished (24–30 pts), having risk of malnutrition (17–23.5 pts), or as malnourished, score ≤17 pts (Guigoz, 2006).

### Mental Well-Being

The Satisfaction with Life Scale (SWLS) was used to assess the subjective well-being perspective of the participants. SWLS measures global cognitive judgments of satisfaction with one’s life. This scale is recommended as a complement to other instruments that focus on psychopathology or emotional well-being because it assesses an individual’s conscious evaluative judgment of his or her life by using the person’s own criteria. The five-item scale results in scores between 1 and 35 pts, with higher values representing higher levels of life’s satisfaction (Laranjeira, 2009). The Happiness Face Scale (HFS) is a pictorial scale used for measuring global subjective happiness related to well-being. The HFS consists of a graphical scheme containing seven faces with different expressions, using a progression of faces from “very happy” to “very sad,” to address the question “How happy are you most of the time?” For each face is assigned one letter (A–G), in which letter A is considered the maximum happiness quotation (with 7 pts) and letter G the minimum value (with 1 pts). The participant will have to identify with one of the faces, depending on their state of happiness (Andrews and Withey, 1976). The Perceived Stress Scale (PSS) is the most widely used instrument for assessing the perception of stress. It is a measure of the level to which situations in one’s life are appraised as stressful. Items were designed to tap how unpredictable, uncontrollable, and overloaded respondents find their lives. The scale also includes a number of direct queries about current levels of experienced stress. Seven out of the 14 items are considered negative and seven as positive. Final scores can vary from 14 to 70 pts, a higher score indicating greater feelings of stress (Trigo et al., 2010). The Centre of Epidemiologic Studies for Depression scale, called CES-D, was also applied. CES-D includes 20 items comprising six sub-scales reflecting major facets of depression: depressed mood, feelings of guilt and worthlessness, feelings of helplessness and hopelessness, psychomotor retardation, loss of appetite, and sleep disturbance (Gonçalves et al., 2014). Responses to each item are given on a four-point Likert scale (0–3) corresponding to the frequency with each symptom was experienced in the past week. Every answer is assigned a score from 0 to 3, respectively. The 20 items total an overall score between 0 and 60, in which the highest scores correlate with more depressive symptoms due to the occurrence frequency of the last week (Ros et al., 2011).

### Functional Fitness

Muscle strength (kg) was measured by HGT using a HD, following the criteria described previously. Gait speed was determined using the 4.6 meters test (GST), which is expressed in seconds. This test consists of the participant walking this distance as quickly as possible. Two trials were performed and the lowest time was used for final scoring (Fried et al., 2001). To assess static balance, the Tandem Stance Balance Test (TSBT) was applied. The TSBT consists of the participant maintaining the standing position with open eyes and one foot in front of the opposite foot for a maximum of 30 s (Cho et al., 2004).

### Exercise Engagement

The exercise adherence was calculated individually (as %) through the total sum of participation. After two consecutive absences, the participant was directly contacted by nursing home to return to the group classes. The minimum adherence accepted for the participant to take part in the study was 60% (exclusion criterion) to minimize bias evidence and in accordance with previous studies (Picorelli et al., 2014). To reduce disparity in data collection, the same evaluators performed the data collection both at baseline and follow-up assessments. The instructor of the sessions did not take part in the data collection processes.

### Data Analysis

The Kolmogorov–Smirnov and visual inspection was done to check the distribution of data. For an older adult population, it should be noted that the intra-individual variability of the data becomes a major research challenge, with regard to homogeneity (Callisaya et al., 2010). Descriptive statistics were summarized as median and standard deviation (M ± SD). Comparisons between groups were performed using *t*-tests for two independent samples. The paired *t*-test accessed differences between variables pre- and post-exercise and percentage-based changes were calculated (Δ%). Linear correlations between all indexes were tested by calculating Spearman’s rho factor. The between-subject mean and SD for each dependent variable was used to convert the changes of all indicators into standardized Cohen effect size (ES). The magnitude of ES was classified following the standards: trivial [*r* ≤ 0.3]; moderate [0.3 < *r* ≤ 0.5]; strong [0.5 < *r* ≤ 0.7], and robust [*r* ≥ 0.7] (Hopkins et al., 2009). The statistical analysis was made with SPSS 20.0 (Statistical Package for Social Sciences, IBM) and *p* ≤ 0.05 used as the level of significance.

## Results

**FIGURE 2:**
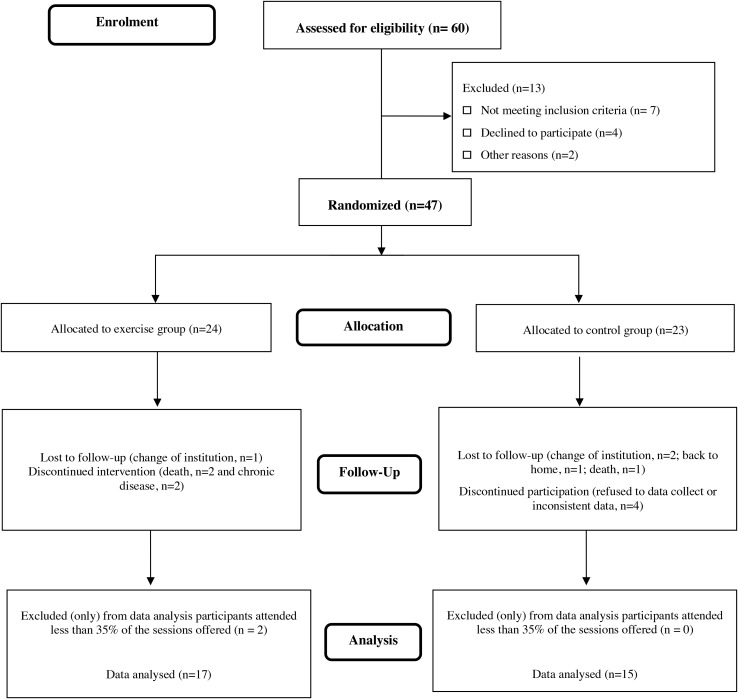
CONSORT flowchart of the study design.

A total of 60 potential participants were initially screened for study admissibility (see flowchart; Figure 2). First, 13 participants were excluded by low interest in taking part of the study (personal decision after the study intervention was explained). Then, 47 older women who matched the inclusion criteria were assigned to the experimental group random division. In total, 13 participants withdrew for several reasons in the follow-up phase and two participants were excluded by low exercise engagement (exclusion criterium 60% adherence). A total of 32 participants (CEP, *n* = 17 and CG, *n* = 15) completed the 14-week study (see Figure 1). No adverse effects were detected as a result of participating in the intervention program. Table 1 shows the baseline characterization of the sample. No statistically significant differences between groups were found. In other words, they were homogeneous for all variables at the beginning of the study.

**TABLE 1:** Anthropometric, nutritional, and clinical characteristics of exercised (CEP) and control (CG) groups at the baseline of a 14-week program of chair-based exercises.

Variables	CEP (*n* = 17)	CG (*n* = 15)	*f* value	*p* value
	M ± SD	M ± SD		
Chronological age (years)	81.17.5	83.38.2	0.63	0.43
Height (m)	1.520.06	1.510.08	1.18	0.29
Weight (kg)	63.210.7	68.717.3	0.16	0.69
Body mass index (kg/m^2^)	27.34.5	30.27.3	1.80	0.19
Charlson Comorbidity Index (score 0–20 points)	7.71.9	8.72.3	1.90	0.18
Mini Nutritional Assessment (score, 0–30 points)	23.72.8	23.82.9	0.00	0.92
Mini-Mental State Examination (score, 0–30 points)	19.24.3	19.35.0	0.00	0.98
Polypharmacy use	6.11.2	5.12.0	0.12	0.76

*Comparison using *t*-test for independent samples.*

*M, mean; SD, Standard deviation; CEP, combined chair-based exercise program; CG, control group non-exercising.*

Table 2 shows the pre- and post-exercise values of the salivary levels of steroid hormones and anti-microbial proteins, MWB, functional fitness, and health status of CEP and CG groups. Significant differences with strong ES were found for levels of salivary TT (*p* < 0.05; *d* = 0.56). The levels of COR showed a statistical tendency to increase in both CEP (*p* = 0.06; *d* = 0.54) and GC groups (*p* = 0.07; *d* = 0.65), with moderate ES. The levels of IgA decreased in the CEP group (*p* < 0.01, *d* = 0.68) with no changes in the CG. No changes were observed for Lys levels in both groups. In the MWB psychometric test of the HFS, significant differences with moderate ES were found for CEP (*p* < 0.05; *d* = 0.30), together with statistical significance with robust ES for the PSS (*p* < 0.01; *d* = 2.60). Significant differences with moderate ES were found for levels of depressive state (*p* < 0.05; *d* = 0.45). Regarding physical fitness tests, statistical differences with robust ES on GST (*p* < 0.05; *d* = 0.60) and TSBT (*p* < 0.05; *d* = 0.80) were observed in the CEP group, whereas no significant alterations were observed in all markers for the GC, except for COR (*p* < 0.05; *d* = 0.65), which showed a significant and moderate increase.

**TABLE 2:** Statistical and effect size scores of pre- and post-intervention comparison of salivary hormones and anti-microbial proteins, mental well-being, functional fitness, and health status of older women.

	CEP (*n* = 17)			Cohen’s *d* effect size	CG (*n* = 15)			Cohen’s *d* effect size
			
	Pre	Post	Δ%		Pre	Post	Δ%	
	M ± SD	M ± SD			M ± SD	M ± SD		
Testosterone (μg/ml)	52.426.3	66.523.6*	+26.8	0.56	61.228.1	52.226.3	−14.7	0.33
Cortisol (μg/ml)	0.200.11	0.260.09	+28.6	0.54	0.200.13	0.310.20*	+55	0.65
Immunoglobulin-A (μg/ml)	262.198.2	151.193.1*	−42.3	0.68	365.292.6	369.185.6	+1	−0.01
Lysozyme (μg/ml)	2.573.98	1.381.60	−46.3	0.39	4.193.44	4.074.77	−2.8	0.01
State of Depression Scale	44.49.1	40.48.3	−9	0.45	37.210.7	35.67.8	−4.3	0.17
Perceived Stress Scale	27.98.0	25.57.7*	−8.6	0.30	28.46.5	28.64.9	+0.7	0.12
Happiness Face Scale	2.51.0	3.40.8**	+36	2.60	3.51.8	2.91.2	−17.1	0.17
Satisfaction with Life Scale	24.95.8	25.56.2	+2.5	0.10	21.77.0	21.16.2	−2.7	0.10
Hand grip strength test	19.611.6	20.410.0	+4	0.07	14.64.4	14.55.0	−0.6	0.01
4.6 meters gait speed test	12.85.2	10.23.2*	−20.3	0.60	18.77.6	17.46.4	−6.9	0.10
Tandem Stance Balance Test	2.67.1	5.99.0*	+123.8	0.80	2.84.9	2.12.0	−25	0.16

**p < 0.01; *p < 0.05.

*M, mean; CEP, combined chair-based exercise program; CG, control group non-exercising; Δ%, percent of variations.*

**FIGURE 3:**
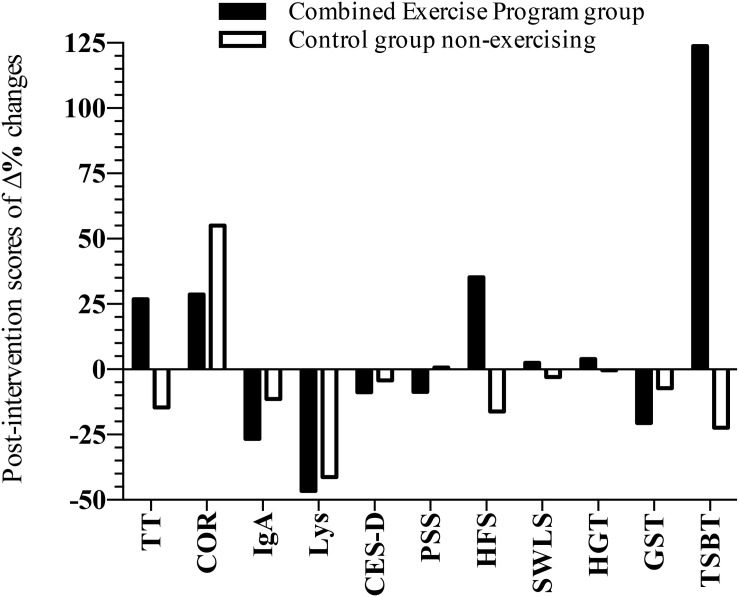
Graphical representation of the pre–post variations (Δ%) of salivary hormones and anti-microbial proteins, MWB, functional fitness, and health status scores of older women after 14 weeks of CEP and non-exercising control group (*n* = 32). COR, cortisol; TT, testosterone; IgA, immunoglobulin-A; Lys, lysozyme; CES-D, Center of Epidemiology Studies for Depression scale; HFS, Happiness Face Scale; PSS, Perceived Stress Scale; SWLS, Satisfaction with Life Scale; TSBT, Tandem Stance Balance Test; HGT, Hand Grip Strength Test; delta percentage scores = [post-value/pre-value]–1.

Results were also expressed as %pre–post variation (Δ%) for all variables after 14 weeks of exercise intervention for CEP group (Figure 3). The TT values decreased 14.6% in the CG, while an increase of 26.8% was observed in the CEP group. On the other hand, higher COR levels were both observed in CEP (+28.6%) and CG groups (+55%). Values of IgA decreased 26% in the CEP and 11% in the CG. Regarding Lys, the values also decreased 46% in the CEP group and 11% in the CG. MWB scores (PSS) presented a slight reduction in the CEP group PSS (−8.6%). Increases of HFS scores were also shown in CEP group (+35.2%), in contrast with the observed decrease of 16.2% in the CG group. Finally, lower GST times (−20.5%) and higher performance from the TSBT test (+123.8%) were found in the CEP group in this pre–post analysis.

Table 3 shows the correlation indexes between the Δ% of all variables analyzed in this study. Salivary COR showed (i) significant (but negative) and moderate correlation with HGT (*r* = −0.48; *p* = 0.00), (ii) a moderate and negative association with TSB (*r* = −0.36; *p* = 0.04), and (iii) a moderately positive association with IgA (*r* = 0.49; *p* = 0.01). The CES-D scale showed a negative and moderate correlation with HFS (*r* = −0.36; *p* = 0.05) and Lys (*r* = −0.42; *p* = 0.02) on the application of the exercise intervention. A negative and moderate association between SWLS and PSS was also found (*r* = −0.35; *p* = 0.05). Moderate and positive correlations between the GST score and HGT (*r* = 0.35; *p* = 0.05), and salivary Lys (*r* = 0.39; *p* = 0.03) scores were observed. In CG, no correlations were significantly observed between any of the Δ% values tested here.

**TABLE 3:** Spearman correlations between pre–post variations (Δ%) in the CEP group of salivary hormones and anti-microbial proteins, mental well-being, functional fitness, and health status scores of older women after 14 weeks of intervention.

Delta percentage score^#^		1	2	3	4	5	6	7	8	9	10
1. Cortisol											
2. Testosterone	*r*	0.303									
	*p*	0.092									
3. State of Depression Scale	*r*	–0.151	–0.008								
	*p*	0.410	0.965								
4. Perceived Stress Scale	*r*	0.237	0.055	0.169							
	*p*	0.191	0.764	0.355							
5. Happiness Face Scale	*r*	–0.137	0.111	−0.356*	–0.069						
	*p*	0.454	0.544	**0.046**	0.708						
6. Satisfaction with Life Scale	*r*	–0.244	–0.107	–0.189	−**0.347**	–0.025					
	*p*	0.179	0.561	0.300	**0.050**	0.891					
7. Hand grip strength test	*r*	−0.483**	–0.205	0.338	0.006	0.078	0.165				
	*p*	**0.005**	0.261	0.058	0.975	0.671	0.367				
8. 4.6 meters gait speed test	*r*	0.177	0.022	–0.193	–0.014	–0.030	–0.095	−0.349*			
	*p*	0.334	0.904	0.291	0.939	0.870	0.605	**0.050**			
9. Tandem Stance Balance Test	*r*	−0.358*	–0.068	–0.123	–0.090	0.300	0.218	0.198	–0.051		
	*p*	**0.044**	0.712	0.502	0.624	0.095	0.231	0.278	0.781		
10. Lysozyme	*r*	0.219	0.042	−0.423*	–0.135	0.083	0.045	−0.390*	–0.043	–0.116	
	*p*	0.229	0.821	**0.016**	0.460	0.652	0.805	**0.027**	0.816	0.528	
11. Immunoglobulin-A	*r*	0.449*	–0.064	–0.219	0.243	–0.192	–0.190	–0.275	**0.342**	–0.027	–0.021
	*p*	**0.010**	0.730	0.228	0.181	0.292	0.297	0.127	**0.050**	0.883	0.907

**p < 0.01; *p < 0.05.

*^#^delta percentage scores = [post-value/pre-value]-1. Bold values represent statistically significant correlations.*

## Discussion

The goals of this study were to verify the effects of CEP in the aging-related health dimensions of functional fitness, subjective well-being, and immune/anti-microbial activity of pre-frail older women. Our main findings indicate that the CEP was capable of improving performance in static balance and gait speed, decreasing feelings of stress, and increasing the state of happiness. The CEP program also showed a clinically relevant immune and anti-microbial response by changing levels of salivary TT, COR, IgA, and Lys. Although the older women participating in this study were properly characterized as pre-frail, it is tempting to suggest that the applied CEP described here, based on salivary biomarkers and well-being/happiness indexes, could also ameliorate physical and mental health of frail older individuals. However, more studies are necessary to address the efficiency of CEP related to the frail stage, when it should be applied, and the physically and mentally progression of the frailty status.

### Salivary-Based Markers

Salivary COR levels are related to psychophysiological systems and they respond to stress stimuli, although the relationship established between COR levels, stress, and well-being is notoriously complex (Kudielka et al., 2009). Therefore, a decrease in salivary levels of COR could reflect a decrease in feelings of stress and, thereby, increased feelings of happiness. However, our results did not show significant correlations between the differences in cortisol levels and the scores from mental health questionnaires. In fact, COR levels increased after the 14-week CEP and this can probably be due to the effect of chronic exercise on the activation of the adrenal glands and stimulation of COR production (Furtado et al., 2016; Ahn and Kim, 2018). Interestingly, CEP participants did show a clear decrease in the stress scale levels together with an increase in the SWLS (see Table 2). COR is also known to stimulate degradation and inhibit synthesis of muscle proteins, contributing to reduced muscle strength, which is therefore associated with loss of physical function (van Schoor et al., 2007). As shown in Table 2, the performance in CEP group was improved in terms of static balance and gait speed. Also, negative correlations between changes (Δ%) in COR and both hand grip strength and static balance were indeed found, which reinforces the conclusion that COR increased levels in CEP are not due to increased stress levels, but represent an adaptation to physical exercise (Hatta et al., 2013). On the other hand, the moderate effect seen on cortisol levels in the CG could point to a poor mental health state because no improvements in those variables were seen.

It is known that a decrease in the concentration of circulating TT in older men is a natural process and possibly serves as a contributing factor to health problems (Harman et al., 2001). Low circulating TT concentration has been associated with cardiovascular disease, reduced cognition, fracture risk, and anemia (Yeap et al., 2018). However, the regular practice of physical exercise can attenuate this process by stimulating TT production and secretion in men and women (Kraemer et al., 2016). In our study, an increase in salivary TT levels was observed in the exercising CEP group (*p* < 0.05; *d* = 0.56), while a trend to decreasing levels was seen in the CG (Table 2). TT can be converted to estradiol and this can benefit many organ systems in female participants, but also directly beneficial effects of TT on female physiology and MWB has been found.

When it comes to the increase of TT after a training period, it is known that an acute hormonal effect (hormone increase) occurs when strength training is performed. However, it appears that the acute response is more pronounced for tissue remodeling than chronic changes, with several studies failing to show a significant variation during muscle strength (hypertrophy) training in the older adults (Kraemer and Ratamess, 2005; Kraemer et al., 2016). Regarding CEP, there are few studies that have evaluated the chronic and acute responses of this type of exercise to TT levels in physically frail elderly (Cadore et al., 2014). Previous studies from our group have shown that both aerobic-like and strength chair-based exercises, applied separately, also brought physical benefits to pre-frail older women, such as agility-dynamic balance, autonomy, lower fear of falling, etc. (Marques et al., 2017). Although not included in that experimental design, we suggest that similar hormonal adjustments to those observed here (TT and COR, Table 2) could have mediated the physical gains observed in a similar study (Rieping et al., 2019).

In addition to the known benefits of increased TT concentration for muscle strength and mass (Paunksnis et al., 2018), a neuroprotective effect is also believed to occur that may affect cognition via androgen and estrogen receptors in the hippocampus, decreasing the neuronal damage caused by oxidative stress and neuronal apoptosis (Yalamanchi and Dobs, 2017). However, the relationship found between endogenous TT and cognitive domains of the main global function such as memory, visuospatial performance, visuo-perceptual, attention, and executive function in healthy older men and women is still conflicting and more studies within this area need to be performed (Hogervorst and Clifford, 2012).

In the last years, few exercise intervention studies have examined salivary antimicrobial proteins, such as IgA and Lys (Allgrove et al., 2008; da Silva et al., 2009; Shibuya et al., 2015). In the study of Akimoto et al. (2003), 12 weeks of regular, moderate, and combined exercises apparently enhanced IgA in older individuals. However, his sample had a much younger average age (almost 20 years younger) and, for this reason, the authors may have observed different results. In another recent long-term intervention study, no changes but a trend toward a moderate increase in IgA secretion was observed after 28 weeks of low-intensity yoga exercises, whereas a trend toward a decrease of IgA secretion was found in the CG (Marques et al., 2017). Intensity of exercise does seem to be an important factor when IgA levels are concerned (Papacosta and Nassis, 2011).

Despite the lack of information regarding the chronic effects of exercise on Lys, some studies showed that Lys, IgA, and other salivary markers, such as alpha-amylase, seem to respond in a similar way during and after physical exercise (Allgrove et al., 2008). These markers share the same control and activation that are regulated by the autonomic nervous system, and are influenced by psychosocial stress (Nater et al., 2006). A negative correlation between the changes in Lys concentration and the CES-D scale did emerge in our study. There are, however, little data available regarding the changes in Lys levels with long-term exercise programs in older frail individuals. A recent study described that Lys secretion increased after moderate-intensity exercise and increased further after high-intensity exercise, which implies that Lys levels may be also related with exercise intensity (Papacosta and Nassis, 2011), and shows the same effects as found for IgA.

### Mental Well-Being

The hypothetical premise that exercise behavior has benefits for subjective psychological well-being still takes place in current scientific discussions (Biedenweg et al., 2014). Many studies have defined subjective well-being as the absence of depressive and anxious symptoms. However, some authors added to this by using subjective perception of happiness and satisfaction with life as a marker of positive well-being (Stubbe et al., 2007).

The results of our study corroborate the recent meta-analysis that indicated that exercise was effective in improving the MWB of older people and that MWB in later life is modifiable through exercise and PA (Windle et al., 2010). In our study, participants exposed to exercise showed increasing levels of positive feelings (happiness and satisfaction with life) and decreased negative feelings (depression and stress). On the other hand, the non-exercise CG showed worse results with a tendency to a decrease in SWLS.

Positive effects of exercise participation have been reported suggesting increases on older adults’ physical self-efficacy that might increase more positive perceptions of subjective well-being and effectively enhancing health-related quality of life (Atlantis et al., 2004; Elavsky et al., 2005; McAuley et al., 2006). The increase in physical functionality in our study may partly justify the results on the psychological side. However, we cannot rule out the influence of the effect of altering the context provided by the exercise program because all participants in the study had never participated in a systematic exercise with specialist teachers and regularity of practice.

Other studies show that systematic PA plays a key role in improving mood states (Oken et al., 2006), self-esteem (Opdenacker et al., 2009; Gothe et al., 2011), and life satisfaction in older adults (Fisher and Li, 2004; Elavsky et al., 2005), all relevant indicators of mental health and well-being. Further evidence for these positive effects of exercise on mental health and well-being in older people have recently been provided as a guideline for PA, and health policies in the United States, offering a strong evidence base for both preventive and therapeutic benefits of regular exercise in improving adult and elderly subjective well-being (National Institute of Aging - U.S, 2018). In European population, a research carried out in 15 countries for the Eurobarometer Study found a strong positive relationship between PA and mental health, revealing the need to implement strategic policies for active, healthy, and participative aging (Abu-Omar et al., 2004).

### Functional Fitness

Our results suggest that CEP attenuated the decrease in functional fitness, even in very aged persons, which can be interpreted as a very positive result, because the physical abilities tested here have a direct connection with their daily life activities. Several studies that assessed multimodal, combined (or multicomponent) exercise-based interventions reported significant improvements in gait speed compared with CGs without exercise (Cadore et al., 2014; Eggenberger et al., 2015). Our results are in accordance with a previous study that concluded that physical exercise, especially when multiple conditioning components are used, is a key factor for the maintenance of the functionality of institutionalized older adults (Cadore et al., 2014).

Emerging evidence suggests that CEP seems to be the most helpful intervention for the prevention of functional decline in people living in social and health care institutions, especially for the preservation or increase of gait speed ability (Giné-Garriga et al., 2010). Recent studies show that low to moderate intensity exercise programs could be enough to develop several functional fitness capacities consistently (Tarazona-Santabalbina et al., 2016). The dose–response relationship between the intensity of the exercises and functional fitness performance was the trademark of the satisfactory results obtained in this study because intensity progression was applied over the 14-week program course.

### Correlations of Δ% Scores

The results of the statistical analysis on correlations confirmed the hypothesis that COR has an association with physical performance. In the study carried out recently, lower levels of diurnal COR were associated with lower levels of global functional fitness, and the opposite was also found regarding highly active subjects (Sousa et al., 2017). That feature led us to raise the hypothesis–also supported by a study from our group (Furtado et al., 2016)–that increasing the levels of COR, to clinically acceptable levels, can benefit the physical–functional performance of older adults.

On the other hand, the negative correlation between the increase of COR and the decrease in IgA promoted by exercise here was in agreement with similar studies already published, including those analyzing Δ% correlations of hormonal markers (Ahn and Kim, 2018). Also, some studies have reported that biological stress can lead to a stressor response at the biological level, causing an immune suppressive effect (Aw et al., 2007). However, many other behavioral factors may have influenced this change.

Other salivary markers, like Lys, also demonstrated significant correlation with psychological (CES-D) and functional physical performance markers (HGT), which may reveal clues about associations between these dimensions. Also, the identified correlations between negative and positive dimensions of MWB and exercise programs suggest that CEP can trigger beneficial psychological effects even in pre-frail old institutionalized populations (Stubbe et al., 2007).

### Limitations

Participants cannot be blinded to group allocation, and we cannot confirm that local and social aspects did not influence the participants’ perception of happiness, well-being, and other psychological status. Our discussion was carried out based on a study with older populations with similar characteristics because comparable previous studies with pre-frail individuals (focusing on the same variables) are still scarce. Other limitations are the number of participants (could be higher) and the fact that only women participated in this study.

### Practical Applications

This study shows that combined CEPs can be safely and easily implemented in older adult populations. The results here (and other similarities) can be extended to a more contemporary approach, through the elaboration of practical application manuals, aimed to inform the benefits of this type of exercises. A good percentage of exercise engagement and significant effects in all dimensions reveal that combined chair-based exercises have high effectiveness in improving the well-being of these populations.

## Conclusion

In conclusion, this study showed that the 14-week CEP program improved functional fitness, subjective well-being, and salivary TT levels in institutionalized older women. Therefore, CEPs could strongly contribute to trigger active behaviors, which could prevent an exponential and early increase of frail individuals in the population.

## Data Availability Statement

The raw data supporting the conclusions of this article will be made available by the authors, without undue reservation.

## Ethics Statement

The studies involving human participants were reviewed and approved by Faculty of Sport Sciences and Physical Education Ethical Committee–University of Coimbra reference code CE/FCDEF-UC/000202013. The patients/participants provided their written informed consent to participate in this study.

## Author Contributions

GF was responsible for data collection and organized the writing of the manuscript. JT, AC, RR, AV-P, and CM helped in the writing of the manuscript. RL performed the statistical analysis. AT and JF coordinated the research, and together with MPB and EH, meticulously reviewed the language and helped with data interpretation. All authors critically revised the article for important intellectual content and approval of the final version.

## Conflict of Interest

The authors declare that the research was conducted in the absence of any commercial or financial relationships that could be construed as a potential conflict of interest.

## References

[B1] Abu-Omar K. Rütten A. Lehtinen V. (2004). Mental health and physical activity in the European Union. *Soz. Und Praventivmed.* 49 301–309. 10.1007/s00038-004-3109-8 15497649

[B2] Ahn N. Kim K. (2018). The effects of resistance elastic bands exercises on salivary iga and salivary cortisol levels in elderly women. *Biomed. Res.* 29 889–894. 10.4066/biomedicalresearch.29-17-2726

[B3] Akimoto T. Kumai Y. Akama T. Hayashi E. Murakami H. Soma R. (2003). Effects of 12 months of exercise training on salivary secretory IgA levels in elderly subjects. *Br. J. Sports Med.* 37 76–79. 12547749 10.1136/bjsm.37.1.76 PMC1724582

[B4] Allgrove J. E. Gomes E. Hough J. Gleeson M. (2008). Effects of exercise intensity on salivary antimicrobial proteins and markers of stress in active men. *J. Sports Sci.* 26 653–661. 10.1080/02640410701716790 18344136

[B5] Andrews F. M. Withey S. B. (1976). “Introduction,” in *Social Indicators of Well-Being*, (Boston, MA: Springer US), 1–24. 10.1007/978-1-4684-2253-5_1

[B6] Artaza-Artabe I. Sáez-López P. Sánchez-Hernández N. Fernández-Gutierrez N. Malafarina V. (2016). The relationship between nutrition and frailty: effects of protein intake, nutritional supplementation, vitamin D and exercise on muscle metabolism in the elderly. A systematic review. *Maturitas* 93 89–99. 10.1016/j.maturitas.2016.04.009 27125943

[B7] Atlantis E. Chow C.-M. Kirby A. Singh M. F. (2004). An effective exercise-based intervention for improving mental health and quality of life measures: a randomized controlled trial. *Prev. Med.* 39 424–434. 10.1016/j.ypmed.2004.02.007 15226056

[B8] Aw D. Silva A. B. Palmer D. B. (2007). Immunosenescence: emerging challenges for an ageing population. *Immunology* 120 435–446. 10.1111/j.1365-2567.2007.02555.x 17313487 PMC2265901

[B9] Barbosa T. M. Marinho D. A. Reis V. M. Silva A. J. Bragada J. A. (2009). Physiological assessment of head-out aquatic exercises in healthy subjects: a qualitative review. *J. Sports Sci. Med.* 8 179–189. 24149524 PMC3761490

[B10] Baumgartner N. Chumlea W. Baumgartner N. Baumgartner N. (1989). Status of anthropometry in elderly subjects3 w composition data. *Am. J. Clin. Nutr.* 50 1158–1166. 2683724 10.1093/ajcn/50.5.1158

[B11] Baylis D. Bartlett D. B. Syddall H. E. Ntani G. Gale C. R. Cooper C. (2013). Immune-endocrine biomarkers as predictors of frailty and mortality: a 10-year longitudinal study in community-dwelling older people. *Age* 35 963–971. 10.1007/s11357-012-9396-8 22388931 PMC3636387

[B12] Biedenweg K. Meischke H. Bohl A. Hammerback K. Williams B. Poe P. (2014). Understanding older adults’ motivators and barriers to participating in organized programs supporting exercise behaviors. *J. Primary Prev.* 35 1–11. 10.1007/s10935-013-0331-2 24214654

[B13] Braga R. (2013). Ética na publicação de trabalhos científicos. *Revista Portuguesa de Medicina Geral e Familiar* 29 354–356.

[B14] Cadore E. L. Casas-Herrero A. Zambom-Ferraresi F. Idoate F. Millor N. Gómez M. (2014). Multicomponent exercises including muscle power training enhance muscle mass, power output, and functional outcomes in institutionalized frail nonagenarians. *Age* 36 773–785. 10.1007/s11357-013-9586-z 24030238 PMC4039263

[B15] Callisaya M. L. Blizzard L. Schmidt M. D. McGinley J. L. Srikanth V. K. (2010). Ageing and gait variability—a population-based study of older people. *Age Age.* 39 191–197. 10.1093/ageing/afp250 20083617

[B16] Cho B. L. Scarpace D. Alexander N. B. (2004). Tests of stepping as indicators of mobility, balance, and fall risk in balance-impaired older adults. *J. Am. Geriatr. Soc.* 52 1168–1173. 15209657 10.1111/j.1532-5415.2004.52317.x

[B17] Clegg A. Young J. Iliffe S. Rikkert M. O. Rockwood K. (2013). Frailty in elderly people. *Lancet* 381 752–762. 10.1016/S0140-6736(12)62167-9 23395245 PMC4098658

[B18] Clegg A. P. Barber S. E. Young J. B. Forster A. Iliffe S. J. (2012). Do home-based exercise interventions improve outcomes for frail older people? Findings from a systematic review. *Rev. Clin. Gerontol.* 22 68–78. 10.1017/S0959259811000165 27226701 PMC4876907

[B19] da Silva R. P. Natali A. J. De Paula S. O. Locatelli J. Marins J. C. B. (2009). Salivary immunoglobulin A (s-lgA) and exercise: relevance of its control in athletes and methodological implications. *Rev, Bras Med. Esporte* 15 459–466. 10.1590/S1517-86922009000700012

[B20] Doody P. Lord J. M. Greig C. A. Whittaker A. C. (2019). Assessing the feasibility and impact of specially adapted exercise interventions, aimed at improving the multi-dimensional health and functional capacity of frail geriatric hospital inpatients: protocol for a feasibility study. *BMJ Open* 9:e031159. 10.1136/bmjopen-2019-031159 31753876 PMC6886909

[B21] Eggenberger P. Theill N. Holenstein S. Schumacher V. de Bruin E. D. (2015). Multicomponent physical exercise with simultaneous cognitive training to enhance dual-task walking of older adults: a secondary analysis of a 6-month randomized controlled trial with I-year follow-up. *Clin. Interv. Aging* 10 1711–1732. 10.2147/CIA.S91997 26604719 PMC4631411

[B22] Elavsky S. McAuley E. Motl R. W. Konopack J. F. Marquez D. X. Hu L. (2005). Physical activity enhances long-term quality of life in older adults: efficacy, esteem, and affective influences. *Ann. Behav. Med.* 30 138–145. 10.1207/s15324796abm3002_6 16173910

[B23] Fisher K. J. Li F. (2004). A community-based walking trial to improve neighbourhood quality of life in older adults: a multilevel analysis. *J. Behav. Med.* 28 186–194. 10.1207/s15324796abm2803_7 15576257

[B24] Fried L. P. Tangen C. M. Walston J. Newman A. B. Hirsch C. Gottdiener J. (2001). Frailty in older adults: evidence for a phenotype. *J. Gerontol.Ser. A Biol. Sci. Med. Sci.* 56 M146–M156. 11253156 10.1093/gerona/56.3.m146

[B25] Furtado G. E. Carvalho H. M. Loureiro M. Patrício M. Uba-Chupel M. Colado J. C. (2020). Chair-based exercise programs in institutionalized older women: salivary steroid hormones, disabilities and frailty changes. *Exp. Gerontol.* 130:110790. 10.1016/j.exger.2019.110790 31816425

[B26] Furtado G. E. Uba-Chupel M. Carvalho H. M. Souza N. R. Ferreira J. P. Teixeira A. M. (2016). Effects of a chair-yoga exercises on stress hormone levels, daily life activities, falls and physical fitness in institutionalized older adults. *Complement. Ther. Clin. Pract.* 24 123–129. 10.1016/j.ctcp.2016.05.012 27502812

[B27] Giné-Garriga M. Guerra M. Pagès E. Manini T. M. Jiménez R. Unnithan V. B. (2010). The effect of functional circuit training on physical frailty in frail older adults: a randomized controlled trial. *J. Aging Phys. Act.* 18 401–424. 10.1123/japa.18.4.401 20956842

[B28] Giné-Garriga M. Roqué-Fíguls M. Coll-Planas L. Sitjà-Rabert M. Salvà A. (2014). Physical exercise interventions for improving performance-based measures of physical function in community-dwelling, frail older adults: a systematic review and meta-analysis. *Arch. Phys. Med. Rehabil.* 95 753.e3–769.e3. 10.1016/j.apmr.2013.11.007 24291597

[B29] Gonçalves B. Fagulha T. Ferreira A. Reis N. (2014). Depressive symptoms and pain complaints as predictors of later development of depression in Portuguese middle-aged women. *Health Care Women Int.* 35 1228–1244. 10.1080/07399332.2013.862795 24279715

[B30] Gothe N. P. Mullen S. P. Wójcicki T. R. Mailey E. L. White S. M. Olson E. A. (2011). Trajectories of change in self-esteem in older adults: exercise intervention effects. *J. Behav. Med.* 34 298–306. 10.1007/s10865-010-9312-6 21222223 PMC3118401

[B31] Grimmer M. Riener R. Walsh C. J. Seyfarth A. (2019). Mobility related physical and functional losses due to aging and disease - A motivation for lower limb exoskeletons. *J. NeuroEng. Rehabil.* 16:2. 10.1186/s12984-018-0458-8 30606194 PMC6318939

[B32] Gruver A. L. Hudson L. L. Sempowski G. D. (2007). Immunosenescence of ageing. *J. Pathol.* 211 144–156. 10.1002/path.2104 17200946 PMC1931833

[B33] Guigoz Y. (2006). The mini nutritional assessment (MNA(registered trademark)) review of the literature - What does it tell us? *J. Nutrit. Health Aging* 10 466–485. 17183419

[B34] Harman S. M. Metter E. J. Tobin J. D. Pearson J. Blackman M. R. (2001). Longitudinal effects of aging on serum total and free testosterone levels in healthy men. *J. Clin. Endocrinol. Metab.* 86 724–731. 10.1210/jcem.86.2.7219 11158037

[B35] Hatta A. Nishihira Y. Higashiura T. (2013). Effects of a single bout of walking on psychophysiologic responses and executive function in elderly adults: a pilot study. *Clin. Interv. Aging* 8 945–952. 10.2147/CIA.S46405 23888111 PMC3722037

[B36] Hek K. Direk N. Newson R. S. Hofman A. Hoogendijk W. J. G. Mulder C. L. (2013). Anxiety disorders and salivary cortisol levels in older adults: a population-based study. *Psychoneuroendocrinology* 38 300–305. 10.1016/j.psyneuen.2012.06.006 22776419

[B37] Hogervorst E. Clifford A. (2012). Exercise to prevent cognitive decline and alzheimer’s disease: for whom, when, what, and (most importantly) how much? *J. Alzheimer’s Dis. Parkinsonism* 02 2–4. 10.4172/2161-0460.1000e117

[B38] Hopkins W. G. Marshall S. W. Batterham A. M. Hanin J. (2009). Progressive statistics for studies in sports medicine and exercise science. *Med. Sci. Sports Exerc.* 41 3–13. 10.1249/MSS.0b013e31818cb278 19092709

[B39] Jadczak A. D. Makwana N. Luscombe-Marsh N. Visvanathan R. Schultz T. J. (2018). Effectiveness of exercise interventions on physical function in community-dwelling frail older people. *JBI Database Syst. Rev. Implement. Rep.* 16 752–775. 10.11124/JBISRIR-2017-003551 29521871

[B40] Kevin A. Louise C. Phillipa L. John G. Tahir M. (2011). Chair based exercise in frail older people: a systematic review. *Eur. Geriatr. Med.* 2:S16. 10.1016/j.eurger.2011.06.003

[B41] Kraemer W. J. Ratamess N. A. (2005). Hormonal responses and adaptations to resistance exercise and training. *Sports Med.* 35 339–361. 10.2165/00007256-200535040-00004 15831061

[B42] Kraemer W. J. Ratamess N. A. Nindl B. C. (2016). Recovery responses of testosterone, growth hormone, and IGF-1 after resistance exercise. *J. Appl. Physiol.* 122 549–558. 10.1152/japplphysiol.00599.2016 27856715

[B43] Kudielka B. M. Hellhammer D. H. H. Wüst S. (2009). Why do we respond so differently? Reviewing determinants of human salivary cortisol responses to challenge. *Psychoneuroendocrinology* 34 2–18. 10.1016/j.psyneuen.2008.10.004 19041187

[B44] Laranjeira C. A. (2009). Preliminary validation study of the Portuguese version of the satisfaction with life scale. *Psychol. Health Med.* 14 220–226. 10.1080/13548500802459900 19235081

[B45] Marques M. Chupel M. U. Furtado G. E. Minuzzi L. G. Rosado F. Pedrosa F. (2017). Influence of chair-based yoga on salivary anti-microbial proteins, functional fitness, perceived stress and well-being in older women: a randomized pilot controlled trial. *Eur. J. Integr. Med.* 12 44–52. 10.1016/j.eujim.2017.04.008

[B46] McAuley E. Konopack J. F. Motl R. W. Morris K. S. Doerksen S. E. Rosengren K. R. (2006). Physical activity and quality of life in older adults: influence of health status and self-efficacy. *Ann. Behav. Med.* 31 99–103. 10.1207/s15324796abm3101_14 16472044

[B47] Miller R. Plessow F. Rauh M. Gröschl M. Kirschbaum C. (2013). Comparison of salivary cortisol as measured by different immunoassays and tandem mass spectrometry. *Psychoneuroendocrinology* 38 50–57. 10.1016/j.psyneuen.2012.04.019 22641005

[B48] Nater U. M. La Marca R. Florin L. Moses A. Langhans W. Koller M. M. (2006). Stress-induced changes in human salivary alpha-amylase activity - Associations with adrenergic activity. *Psychoneuroendocrinology* 31 49–58. 10.1016/j.psyneuen.2005.05.010 16002223

[B49] National Institute of Aging - U.S (2018). *Exercise & Physical Activity: Your everyday guide.* Washington, DC: Department of Health and Human Services.

[B50] Nelson M. E. Rejeski W. J. Blair S. N. Duncan P. W. Judge J. O. King A. C. (2007). Physical activity and public health in older adults: recommendation from the American College of Sports Medicine and the American Heart Association. *Med. Sci. Sports Exerc.* 39 1435–1445. 10.1249/mss.0b013e3180616aa2 17762378

[B51] Oken B. S. Zajdel D. Kishiyama S. Flegal K. Dehen C. Haas M. (2006). Randomized, controlled, six-month trial of yoga in healthy seniors: effects on cognition and quality of life. *Altern. Ther. Health Med.* 12 40–47. 10.1073/pnas.111134598 16454146 PMC1457100

[B52] Opdenacker J. Delecluse C. Boen F. (2009). The longitudinal effects of a lifestyle physical activity intervention and a structured exercise intervention on physical self-perceptions and self-esteem in older adults. *J. Sport Exerc. Psychol.* 31 743–760. 20384010 10.1123/jsep.31.6.743

[B53] Papacosta E. Nassis G. P. (2011). Saliva as a tool for monitoring steroid, peptide and immune markers in sport and exercise science. *J. Sci. Med. Sport* 14 424–434. 10.1016/j.jsams.2011.03.004 21474377

[B54] Park B. J. Lee Y. J. (2010). Integrative approach to elderly frailty. *Korean J. Fam. Med.* 31 747–754. 10.4082/kjfm.2010.31.10.747

[B55] Paunksnis M. R. Evangelista A. L. La Scala Teixeira C. V. Alegretti João G. Pitta R. M. Alonso A. C. (2018). Metabolic and hormonal responses to different resistance training systems in elderly men. *Aging Male* 21 106–110. 10.1080/13685538.2017.1379489 28937309

[B56] Petrini C. (2014). Helsinki 50 years on. *La Clinica Terapeutica* 165 179–181. 25203329 10.7417/CT.2014.1729

[B57] Picorelli A. M. A. Pereira L. S. M. Pereira D. S. Felício D. Sherrington C. (2014). Adherence to exercise programs for older people is influenced by program characteristics and personal factors: a systematic review. *J. Physiother.* 60 151–156. 10.1016/j.jphys.2014.06.012 25092418

[B58] Quan H. Li B. Couris C. M. Fushimi K. Graham P. Hider P. (2011). Updating and validating the Charlson comorbidity index and score for risk adjustment in hospital discharge abstracts using data from 6 countries. *Am. J. Epidemiol.* 173 676–682. 10.1093/aje/kwq433 21330339

[B59] Rathleff C. R. Bandholm T. Spaich E. G. Jorgensen M. Andreasen J. (2017). Unsupervised progressive elastic band exercises for frail geriatric inpatients objectively monitored by new exercise-integrated technology-a feasibility trial with an embedded qualitative study. *Pilot Feasibility Stud.* 3:56. 10.1186/s40814-017-0202-3 29158914 PMC5683376

[B60] Révész D. Verhoeven J. E. Milaneschi Y. De Geus E. J. C. N. Wolkowitz O. M. Penninx B. W. J. H. (2014). Neurobiology of Aging Dysregulated physiological stress systems and accelerated cellular aging. *Neurobiol. Aging* 35 1422–1430. 10.1016/j.neurobiolaging.2013.12.027 24439483

[B61] Rhebergen D. Korten N. C. M. Penninx B. W. J. H. Stek M. L. Van Der Mast R. C. Voshaar R. O. (2015). Hypothalamic — pituitary — adrenal axis activity in older persons with and without a depressive disorder. *Psychoneuroendocrinology* 51 341–350. 10.1016/j.psyneuen.2014.10.005 25462906

[B62] Rieping T. Furtado G. E. Letieri R. V. Chupel M. U. Colado J. C. Hogervorst E. (2019). Effects of different chair-based exercises on salivary biomarkers and functional autonomy in institutionalized older women. *Res. Q. Exerc. Sport* 90 1–10. 10.1080/02701367.2018.1563272 30722757

[B63] Ros L. Latorre J. M. Aguilar M. J. Serrano J. P. Navarro B. Ricarte J. J. (2011). Factor structure and psychometric properties of the center for epidemiologic studies depression scale (CES-D) in older populations with and without cognitive impairment. *Int. J. Aging Hum. Dev.* 72 83–110. 21639012 10.2190/AG.72.2.a

[B64] Ruan Q. Yu Z. Chen M. Bao Z. Li J. He W. (2015). Cognitive frailty, a novel target for the prevention of elderly dependency. *Ageing Res. Rev.* 20 1–10. 10.1016/j.arr.2014.12.004 25555677

[B65] Shephard R. J. (2002). Ethics in exercise science research. *Sports Med.* 32 169–183. 11839080 10.2165/00007256-200232030-00002

[B66] Shibuya T. Kaburagi T. Nagai R. Oshiro S. (2015). The effects of moderate exercise on secretory IgA production in mice depends on dietary carbohydrate intake. *J. Clin. Biochem. Nutr.* 57 44–49. 10.3164/jcbn.15-21 26236100 PMC4512897

[B67] Sousa A. C. P. de A. Marchand A. Garcia A. Gomez J. F. Ylli A. (2017). Cortisol and physical performance in older populations: findings from the international mobility in aging study (IMIAS). *Arch. Gerontol. Geriatr.* 71 50–58. 10.1016/j.archger.2017.03.002 28343089

[B68] Stubbe J. H. de Moor M. H. M. Boomsma D. I. de Geus E. J. C. (2007). The association between exercise participation and well-being: a co-twin study. *Prev. Med.* 44 148–152. 10.1016/j.ypmed.2006.09.002 17059845

[B69] Syddall H. Cooper C. Martin F. Briggs R. Aihie Sayer A. (2003). Is grip strength a useful single marker of frailty? *Age Age.* 32 650–656. 10.1093/ageing/afg111 14600007

[B70] Tanaka H. Monahan K. D. Seals D. R. (2001). Age-predicted maximal heart rate revisited. *J. Am. College Cardiol*. 37, 153–156. 10.1016/s0735-1097(00)01054-8 11153730

[B71] Tarazona-Santabalbina F. J. Gómez-Cabrera M. C. Pérez-Ros P. Martínez-Arnau F. M. Cabo H. Tsaparas K. (2016). A multicomponent exercise intervention that reverses frailty and improves cognition, emotion, and social networking in the community-dwelling frail elderly: a randomized clinical trial. *J. Am. Med. Dir. Assoc.* 17 426–433. 10.1016/j.jamda.2016.01.019 26947059

[B72] Teixeira A. M. Ferreira J. P. Hogervorst E. Braga M. F. Bandelow S. Rama L. (2016). Study protocol on hormonal mediation of exercise on cognition, stress and immunity (PRO-HMECSI): effects of different exercise programmes in institutionalized elders. *Front. Public Health* 4:133. 10.3389/fpubh.2016.00133 27446898 PMC4921497

[B73] Travers J. Romero-Ortuno R. Bailey J. Cooney M. T. (2019). Delaying and reversing frailty: a systematic review of primary care interventions. *Br. J. Gen. Pract.* 69 E61–E69. 10.3399/bjgp18X700241 30510094 PMC6301364

[B74] Trigo M. Canudo N. Branco F. Silva D. (2010). Estudo das propriedades psicométricas da perceived stress scale (PSS) na população portuguesa. *Phychologica* 53 353–358.

[B75] van Schoor N. M. Knol D. L. de Ronde W. Lips P. Eekhoff E. M. W. Visser M. (2007). Relationship between cortisol and physical performance in older persons. *Clin. Endocrinol.* 67 398–406. 10.1111/j.1365-2265.2007.02900.x 17555515

[B76] Walsh N. P. Gleeson M. M. Shephard R. J. Jeffrey M. G. Woods A. Bishop N. C. (2011). Position statement part one: immune function and exercise. *Exerc. Immunol. Rev.* 17 6–63. 21446352

[B77] Windle G. Hughes D. Linck P. Russell I. Woods B. (2010). Is exercise effective in promoting mental well-being in older age? A systematic review. *Aging Ment. Health* 14 652–669. 10.1080/13607861003713232 20686977

[B78] Yalamanchi S. Dobs A. (2017). Debate position: cognition and mood are not improved in men administered exogenous testosterone therapy. *Curr. Opin. Urol.* 27 525–531. 28863017 10.1097/MOU.0000000000000435

[B79] Yeap B. B. Page S. T. Grossmann M. (2018). Testosterone treatment in older men: clinical implications and unresolved questions from the Testosterone Trials. *Lancet Diabetes Endocrinol.* 6 659–672. 10.1016/S2213-8587(17)30416-3 30017800

